# Depicting the Non-Covalent Interaction of Whey Proteins with Galangin or Genistein Using the Multi-Spectroscopic Techniques and Molecular Docking

**DOI:** 10.3390/foods8090360

**Published:** 2019-08-23

**Authors:** Chun-Min Ma, Xin-Huai Zhao

**Affiliations:** Key Laboratory of Dairy Science, Ministry of Education, Northeast Agricultural University, Harbin 150030, China

**Keywords:** galangin, genistein, whey protein isolate, spectroscopy, molecular docking

## Abstract

The non-covalent interactions between a commercial whey protein isolate (WPI) and two bioactive polyphenols galangin and genistein were studied at pH 6.8 via the multi-spectroscopic assays and molecular docking. When forming these WPI-polyphenol complexes, whey proteins had changed secondary structures while hydrophobic interaction was the major driving force. Detergent sodium dodecyl sulfate destroyed the hydrophobic interaction and thus decreased apparent binding constants of the WPI-polyphenol interactions. Urea led to hydrogen-bonds breakage and protein unfolding, and therefore increased apparent binding constants. Based on the measured apparent thermodynamic parameters like ΔH, ΔS, ΔG, and donor-acceptor distance, galangin with more planar stereochemical structure and random B-ring rotation showed higher affinity for WPI than genistein with location isomerism and twisted stereochemical structure. The molecular docking results disclosed that β-lactoglobulin of higher average hydrophobicity had better affinity for the two polyphenols than α-lactalbumin of lower average hydrophobicity while β-lactoglobulin possessed very similar binding sites to the two polyphenols. It is concluded that polyphenols might have different non-covalent interactions with food proteins, depending on the crucial polyphenol structures and protein hydrophobicity.

## 1. Introduction

Flavonoids are important naturally polyphenols distributed in the plants, and have received considerable attention in the recent years because flavonoids have anti-oxidant, anti-cancer, and anti-inflammatory activities [1]. Flavonoids have both benzopyran (the A- and C-rings) and phenyl (the B-ring) structures, and are categorized into several classes including isoflavones, flavonols, flavones, anthocyanidins, and others, based on B-ring position and C-ring variation. Galangin belongs to flavones and is derived from the *Alpinia officinarum* (a traditional medicinal plant) root, and is an active component in propolis [2]. Genistein is one member of isoflavones, and is particular abundant in soybean (*Glycine max* (Linn.) Merr.) and its processed products [3]. In the past studies, both galangin and genistein had been assessed for their anti-oxidant, anti-tumor, anti-inflammatory effects and their prevention on cardiovascular disease [2,3,4].

Proteins are important nutritional components in the dietary foods, and can interact with other dietary components such as flavonoids. Flavonoids interact with proteins either non-covalently or covalently [5], leading to the formation of protein-flavonoid complexes and conjugates. Various proteins such as α-lactalbumin, lactoferrin, casein, and soybean proteins have been assessed for their interactions with several flavonoids [6,7,8,9]. Quercetin and tea polyphenols are the most studied polyphenols due to their abundant existence in plant foods. Quercetin and its metabolites can complex non-covalently with bovine serum albumin (BSA), human serum albumin, and rat serum albumin under physiological conditions [10], and also can bind with soybean proteins covalently at pH 9 [11]. Tea polyphenols can interact with whole milk proteins [12], casein [13], individual milk proteins (e.g., β-lactoglobulin, α-casein, or β-casein) [14,15], and other proteins [16]. However, two bioactive polyphenols galangin and genistein have not been well-studied yet for their interactions with a commercial protein ingredient whey protein isolate (WPI).

These protein-flavonoid interactions may induce changed functions, structures, and nutritional properties for proteins. The binding of apigenin, kaempferol, quercetin, and myricetin to soybean proteins induced decreased protein digestibility and changed protein secondary or tertiary structures [11]. Moreover, the protein-flavonoid interactions might lead to decreased health functions for flavonoids [17]. When quercetin, rutin, and catechins interacted with plasma proteins, they showed decreased activities to scavenge the 2,2’-azinobis-3-enthylbenzothiazoline-6-sulfonic acid (ABTS) radicals [18]. Casein addition brought about decreased growth inhibition of epigallocatechin gallate (EGCG) in colon cancer HT29 cells because of the casein-EGCG interaction [19]. In the presence of whey proteins, the anti-microbial activities of different Argentinean green tea were reduced [20]. However, the protein-polyphenol interactions were evidenced in a previous study to increase the thermal stability of both fisetin and quercetin in solutions [21].

As one of important protein ingredients, WPI has various applications in the food industry. Such investigation about the non-covalent interactions between WPI and galangin/genistein thus deserves our consideration. In this study, the WPI-galangin or WPI-genistein interactions were assessed using multi-spectroscopic techniques, and were verified using sodium dodecyl sulfate (SDS) or urea to interfere with these chemical forces involved in the protein-polyphenol interactions. Subsequently, possible polyphenol binding sites in two whey protein fractions α-lactalbumin and β-lactoglobulin as well as the interaction energy were estimated using the molecular docking. This study aimed to reveal the crucial roles of polyphenol structures and protein hydrophobicity for these interactions.

## 2. Materials and Methods

### 2.1. Materials

The commercial bovine WPI with protein content of 900.0 g/kg (dry basis) was obtained from Davisco Food International Inc. (Le Sueur, MN, USA). Galangin and genistein with 98% purity were bought from Meilun Biotechnology Co. Ltd. (Dalian, China). SDS and urea were purchased from Sigma-Aldrich Co. (St. Louis, MO, USA) and Xilong Chemical Industry Co. Ltd. (Shantou, China), respectively. All other chemicals were analytical reagents, while the water used was distilled water.

### 2.2. Preparation of Sample Solutions

WPI was dissolved in 50 mmol/L phosphate buffer solution (PBS, pH 6.8) to obtain a final protein concentration of 20 μmol/L, using an estimated average molecular weight of 17 kDa for WPI. Both galangin and genistein were dispersed separately in anhydrous ethanol to yield respective stock solutions of 1 mmol/L, and kept in the dark at 4 °C before use. The stock polyphenol solutions were mixed with the WPI solution to obtain serial polyphenol concentrations (5–40 μmol/L) with final ethanol concentration less than 1%. Moreover, to verify the effects of SDS and urea on the WPI-polyphenol interaction, SDS and urea (dissolved in the PBS) were also added to the reaction systems at final levels of 5 mmol/L and 4 mol/L, respectively. After a reaction time of 30 min at three temperatures (293, 303, and 313 K), the mixed solutions were transferred into quartz glass cuvettes to measure their fluorescence intensity values.

### 2.3. Assays of Fluorescence Spectroscopy

Fluorescence spectrum of the WPI solutions with or without galangin, genistein, SDS, and urea were measured at the F-4500 fluorescence spectrophotometer (Hitachi, Kyoto, Japan). The excitation wavelength was set at 280 nm. The emission scan was recorded from 300 to 450 nm using scan speed of 240 nm/min. The slit widths of both excitation and emission were fixed at 5 nm.

#### 2.3.1. Determination of Fluorescent Quenching Mechanism

Fluorescence quenching, as a process of decreasing fluorescence intensity of a given fluorophore, is regarded through two mechanisms (static and dynamic quenching) [22]. The Stern–Volmer equation below (Equation (1)) was used to elucidate the fluorescence quenching mechanisms.

F0/F = 1 + Ksv·Q = 1 + Kq·δ0·Q,(1)

where, F and F0 are detected fluorescence intensity values with or without the quencher (galangin or genistein), respectively. Q is quencher concentration, while δ_0_ is the bimolecular fluorescence life-time (10^−8^ s) without quencher [23]. Ksv is the Stern–Volmer quenching rate constant. Kq is the quenching rate constant of the bimolecular. When Kq value is larger than 2 × 10^10^ L/(mol·s), the reduced fluorescence intensity is regarded to be caused by a static quenching; otherwise, a dynamic quenching occurs for the quencher-protein binding [23].

#### 2.3.2. Assays of Apparent Binding Constants and Site Numbers

In the static quenching, the small molecules bind independently to a set of equivalent sites of a macromolecule [8]. The apparent binding constant (Ka) and binding site numbers (n) for the WPI-polyphenol interactions were obtained using the plot of lg[(F0−F)/F] vs. lgQ, according to the Equation (2).

lg[(F0 − F)/F] = lgK + n·lgQ,(2)

where, F and F0 are the respective fluorescence intensity values with or without quencher, while Q is quencher concentration.

#### 2.3.3. Assay of Apparent Thermodynamic Parameters

Apparent enthalpy and entropy changes (ΔH and ΔS) were obtained from the slope and intercept of the curve of lgKa against 1/T based on the Vaan’t Hoff equation (Equation (3)), while apparent free energy change (ΔG) was estimated using the Gibbs–Helmholtz equation (Equation (4)) [24].

In Ka = −ΔH/(RT) + ΔS/R,(3)

ΔG = ΔH − TΔS,(4)

where, R is gas constant [8.314 J/(mol K)] and T refers to the used temperature. Ka is the binding constant at a set temperature. In general, four non-covalent interaction forces are involved in the non-covalent interactions between small molecules and bio-molecules. When ΔH > 0 and ΔS > 0, hydrophobic force is the main driving force; if ΔH < 0 and ΔS < 0, hydrogen-bonds and van der Waals force are the dominant forces; however, if ΔH < 0 and ΔS > 0, electrostatic force governs the interaction [5].

#### 2.3.4. Assay of Efficiency of Energy Transfer

Efficiency of energy transfer was estimated using the Förster non-radiative energy transfer theory [22]. Energy transfer efficiency (E) was thus calculated using the Equation (5).

E = 1 − F/F0 = R_0_^6^/(R_0_^6^ + r^6^), (5)

where, F and F0 represent the respective fluorescence intensity values with or without the quencher. R_0_ is the critical distance that 50% of the energy is transferred to proteins, while r is the distance of the donor and acceptor. R_0_ is given by another equation as below (Equation (6)).

R_0_^6^ = 8.8 × 10^−25^·K^2^·N^−4^·φ·J, (6)

where, K^2^ is a spatial orientation factor; N is the refractive index of medium; φ is the fluorescence quantum yield of the donor; J is the overlap integral that expresses the effect of overlap between the emission spectrum of the donor and the absorption spectrum of the acceptor, and can be calculated using Equation (7).

J = ∑F(λ)·ε(λ)·λ^4^·dλ/(∑F(λ)·dλ), (7)

where, F(λ) is the fluorescence intensity value of the donor at wavelength λ, while ε(λ) is the molar absorption coefficient of the acceptor at wavelength λ. The equations of F(λ) and ε(λ) were acquired using a non-linear curve fitting.

### 2.4. Assay of Ultra-Violet Spectroscopy

The ultra-violet (UV) spectra were assayed using a UV-2600 UV-Vis spectrophotometer (Shimadzu, Kyoto, Japan). The WPI solution of 20 μmol/L was mixed with the stock polyphenol solutions to receive various polyphenol concentrations (5–40 μmol/L), and then kept at 293 K for 30 min. The resultant mixtures were measured for absorption intensity values at 250–400 nm with a sampling interval of 0.1 nm. The PBS was used to adjust baseline for this assay.

### 2.5. Assay of Three-Dimensional Fluorescence Spectra

3D fluorescence spectra of the WPI solution at 293 K were assayed with or without equimolar polyphenol concentration at the fluorescence spectrophotometer (Type F-4500, Hitachi, Kyoto, Japan), using a scanning rate of 240 nm/min. The respective emission and excitation wavelengths were used at 210–400 and 210–350 nm. Other parameters were the same to those used in the assay of fluorescence spectra.

### 2.6. Molecular Docking

The crystal structures of β-lactoglobulin (variant A, PDB ID: 2Q2M) and α-lactalbumin (PDB ID: 1HFX) were obtained from the Protein Data Bank (https://www.rcsb.org) [25,26]. The 3D structures of galangin and genistein were downloaded from the NCBI database of PubChem (Bethesda, MD, USA). The molecular docking was done and calculated using the AutoDock 4.2 package (Scripps Institution, San Diego, CA, USA). AutoDockTools version 1.5.4 in this package was used to prepare the ligands and receptors by adding hydrogen atoms and charges, removing water molecules before molecular docking. The value of grid box and gird space were set as 6 × 6 × 6 nm and 0.0375 nm, respectively. Possible binding sites of the ligands in proteins were calculated using the Lamarckian Genetic Algorithm [27]. The numbers of genetic algorithm runs were 100, while the conformers with the lowest interaction energy were selected for further analysis. Docking results were visualized using the Discovery Studio 3.0 Visualizer program (Accelrys Co., San Diego, CA, USA).

### 2.7. Statistical Analysis

All experiments or assays were repeated three times and reported data were means or means ± standard deviations. The non-linear curve fitting was performed using the Origin 8.0 (OriginLab Co., Northampton, MA, USA). Statistical analysis was performed using the SPSS 17.0 software (SPSS Inc., Chicago, IL, USA). One-way analysis of variance (ANOVA) was used, while the differences between the means were analyzed by Duncan’s multiple range tests at a significance level of 0.05.

## 3. Results

### 3.1. The Non-Covalent Interactions between WPI and Galangin or Genistein

**Figure 1:**
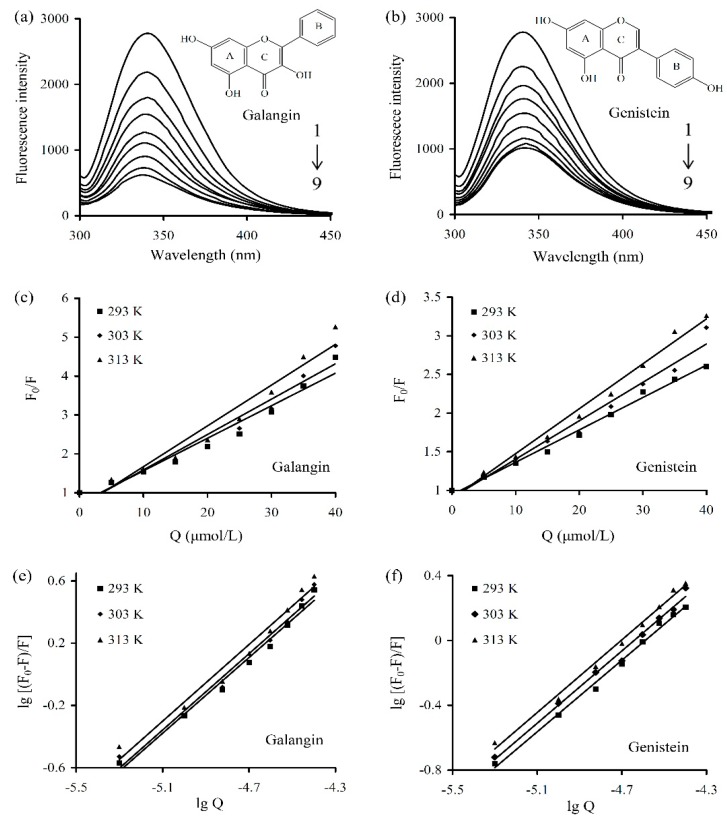
The fluorescence spectrum (300–450 nm) of whey protein isolate (WPI) solution with or without galangin (**a**) and genistein (**b**) at 293 K, the Stern-Volmer plots for galangin (**c**) or genistein (**d**), as well as the lg[(F0 − F)/F] vs. lgQ plots for the binding of galangin (**e**) or genistein (**f**) with WPI at the three temperatures. WPI was used at 20 μmol/L, while galangin/genistein was used at 0, 5, 10, 15, 20, 25, 30, 35, and 40 μmol/L (from 1 to 9).

The fluorescence spectra of the prepared WPI solution containing various levels of galangin and genistein are shown in Figure 1a,b. Fluorescence intensity value of the WPI solution decreased as polyphenol concentration increased, indicating the formation of WPI-polyphenol complexes and fluorescence quenching of the two polyphenols. The results describing the linear Sterne Volmer plots of F0/F against Q are shown in Figure 1c,d. Moreover, all measured Kq values (Table 1) were larger than 2 × 10^10^ L/(mol s), demonstrating a static quenching mechanism.

Further data treatment (i.e., lg[(F0 − F)/F] vs. lgQ) (Figure 1e,f) yielded the values of Ka and n at the three temperatures (Table 2). In total, the n values of the studied interactions were close to 1, indicating there was only a single binding site in WPI available for the two polyphenols. The Ka values for the WPI-galangin interaction were higher than those of the WPI-genistein interaction [(6.96 − 9.64) × 10^5^ vs. (1.08 − 3.18) × 10^5^ L/mol] at these temperatures, suggesting that WPI had higher affinity for galangin other than genistein. However, when SDS (or urea) was added into the WPI solution, the assessed WPI-polyphenol interactions were impacted. The results showed (Table 3) that the Ka values for the WPI-galangin and WPI-genistein interactions at 293 K in the presence of SDS were decreased to corresponding 2.24 × 10^5^ and 3.93 × 10^4^ L/mol, showing SDS’s ability to damage the WPI-polyphenol interactions. Moreover, the Ka values for the two interactions at 293 K in the presence of urea were increased to 8.95 × 10^5^ and 1.59 × 10^5^ L/mol, respectively, demonstrating urea’s ability to promote the WPI-polyphenol interactions.

Other apparent thermodynamic parameters such as the enthalpy, entropy, and the most important free energy changes (ΔH, ΔS, and ΔG) were also calculated (Table 2). The ΔG values for the WPI-galangin interaction at the three temperatures were 32.8, 34.4, and 35.9 kJ/mol, whilst those for the WPI-genistein interaction were 28.1, 30.5, and 32.9 kJ/mol, respectively, revealing that these interactions were a spontaneous non-covalent binding process. Also, the WPI-galangin interaction was stronger than the WPI-genistein interaction, because the first one had more negative ΔG values. Both ΔH and ΔS measured in this study had positive values. The major force involved in these interactions thus was the hydrophobic interaction.

**Figure 2:**
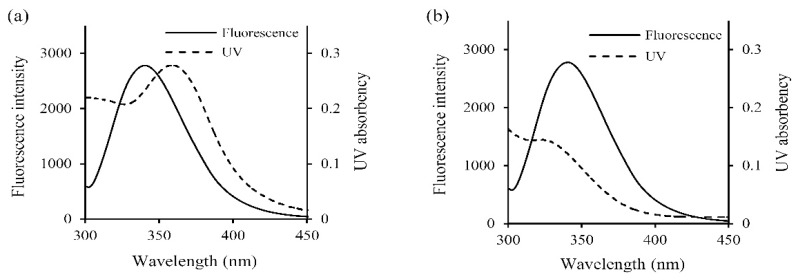
Overlap features of the fluorescence emission spectra of WPI and ultra-violet (UV) absorption spectra of galangin (**a**) or genistein (**b**). Both WPI and galangin/genistein were used at 20 μmol/L.

Fluorescence quenching occurred in donor (WPI) indicated that there was energy transfer to the two acceptors (galangin and genistein). Based on the Förster’s non-radiative energy transfer theory (Figure 2), the present study employed tryptophan (Trp) fluorescence quantum yield (φ value) of 0.2, random orientation (K^2^ value) of 2/3, and refractive index of medium water (n, average value) of 1.336 as the previous research did [22]. As shown in Table 4, the distances (r values) of the donor WPI and two acceptors in the present assay were all less than 7 nm, suggesting an energy transfer occurred with high probability.

### 3.2. Secondary Conformation Changes of WPI Induced by the Non-Covalent Interactions

**Figure 3:**
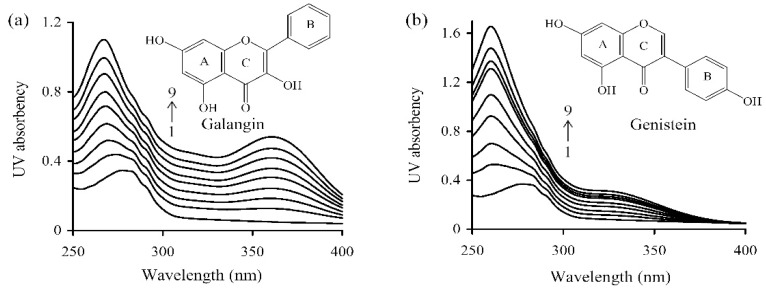
UV absorption spectrum of WPI solution without or with galangin (**a**) or genistein (**b**). WPI was used at 20 μmol/L, while galangin/genistein was used at 0, 5, 10, 15, 20, 25, 30, 35, and 40 μmol/L (from 1 to 9).

UV-absorption spectra changes of WPI solution under various galangin/genistein levels were shown in Figure 3. The two polyphenols led to blue-shifted UV-absorption for WPI solution, because the detected wavelengths of the absorption peak decreased as galangin/genistein concentration increased. In the presence of galangin (or genistein) of 40 μmol/L, the absorption peak of WPI solution shifted from 277 to 260 (or 267 nm). This indicated that the binding of galangin and genistein with WPI led to changed protein skeleton or conformation.

To further depict the WPI-polyphenol interactions, the 3D fluorescence spectra technique was used to measure corresponding changes in both peak position and peak intensity values. In general, the peak I (λ_ex_ near 240 nm) mainly reflects the spectral features of polypeptide backbones or the secondary structures of proteins, while peak II is used to show spectral behaviors of tyrosine (Tyr) and Trp residues in proteins. The results (Table 5) pointed out that WPI had changed secondary conformation, because both galangin and genistein led to shifted wavelengths for both peak I and peak II (λ_em_ values increased from 340 to 345 nm). It was evident again that the non-covalent interactions between WPI and the two polyphenols brought about unfolded conformation for whey proteins, exposure of Tyr and Trp, and thereby decreased fluorescence intensity values (from 2327 to 639.6 and 1028).

### 3.3. The Binding Sites and Interaction Energy of the WPI-Polyphenol Interactions

**Figure 4:**
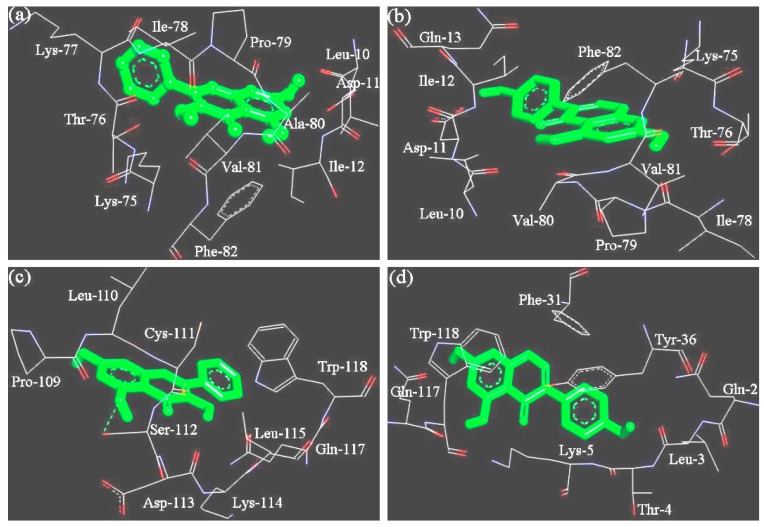
The docked sites for the non-covalent binding of β-lactoglobulin (**a**,**b**) or α-lactalbumin (**c**,**d**) with galangin (**a**,**c**) or genistein (**b**,**d**). Hydrogen-bonds are shown by green dashes.

Bovine whey proteins contain two major proteins (α-lactalbumin and β-lactoglobulin) and other minor proteins [24]. Thus, α-lactalbumin and β-lactoglobulin (variant A) were selected as model proteins to dock with respective galangin and genistein, and then were estimated for the interaction energy and possible binding sites. The docking results (Figure 4 and Table 6) indicated that α-lactalbumin possessed binding sites different from the β-lactoglobulin for the two polyphenols. The hydrophobic pockets of the β-lactoglobulin to galangin and genistein were only different in one amino acid residue (Lys-77 vs. Gln-13). Both galangin and genistein thus were bound into the β-lactoglobulin via very similar binding site. However, α-lactalbumin provided different binding sites to galangin and genistein. Moreover, the results also indicated that the β-lactoglobulin had higher interaction energy than α-lactalbumin when the two proteins were docked with the same polyphenol molecule (Table 6). The interaction energy values of β-lactoglobulin with the two polyphenols (Table 6) were basically consistent with the measured free energy changes using the spectroscopic technique (Table 2). Thus, the β-lactoglobulin has stronger interaction with galangin and genistein, compared with α-lactalbumin.

It is shown in the 3D docking mode (Figure 4) that several hydrophobic amino acid residues in the protein molecules provided an essential stabilization for these protein-polyphenol complexes, via the crucial hydrophobic interaction. Hydrophobic interaction thus played key role in the formation of these protein-polyphenol complexes.

## 4. Discussion

The fluorescence assay was a widely accepted technique for the determination of the non-covalent interactions between proteins and small molecules, because of its high sensitivity, ease of implementation, and rapidity [28]. Fluorescence assay not only provides considerable binding information, but also allows the measurement of the association reactions under low substance concentration and various conditions. In this study, the fluorescence of WPI in solution was quenched by the target molecules galangin and genistein. This phenomenon was consistent with that observed in a previous study investigating the interactions between four flavonoids (apigenin, naringenin, kaempferol, and genistein) and β-lactoglobulin [23]. The decrease in fluorescence intensity of protein is partly caused by the change of protein conformation and exposure of the hydrophobic amino acid residues (mainly Trp) to the solvents [29]. Fluorescence quenching also means the transferring of the quenchers from the polar aqueous solution to the hydrophobic pockets of proteins [6]; thereby, these quenchers reduces fluorescence quantum yield of Trp and other amino acid residues and induces a decrease in fluorescence intensity for the proteins. When resveratrol was bound to the Trp and Tyr residues in trypsin, the yielded non-covalent interaction directly brought about fluorescence quenching of trypsin [30]. When curcumin was mixed with β-casein, the resultant complex formation reduced fluorescence intensity of the Trp residues [31]. It is thus reasonable that the present fluorescence assay results proved the inclusion of galangin and genistein in the hydrophobic pockets of the whey proteins in WPI. These results (Table 4) also illustrated that there occurred energy transfer and the interaction between WPI and galangin (or genistein).

In general, the hydrogen-bonds and hydrophobic interaction are two key non-covalent forces involved in the protein–phenol interactions. For example, apigenin and naringenin could be bound to β-lactoglobulin with the hydrogen-bonds [32]; however, the interactions between two polyphenols (quercetin and green tea polyphenol) and two proteins (soybean protein and α-lactalbumin) were mainly achieved by the hydrophobic interactions [8,9]. In this study, the non-covalent interactions between WPI and the two polyphenols were also mediated by this hydrophobic interaction (Table 2). When galangin or genistein molecules interacted with the whey proteins in WPI, the hydrophobic hydration of these proteins was destroyed. Subsequently, some bound water molecules were released into the medium and then existed as free water molecules; the whole process thereby showed positive ΔS values, from a chemical point of view [33]. To verify the detected non-covalent WPI-galangin and WPI-genistein interactions, two compounds urea and SDS were also used. Urea as a hydrogen-bonds receptor is powerful to damage the hydrogen-bonds in proteins and thereby causes protein unfolding, while SDS as an anionic detergent can partly or totally destroy the hydrophobic interaction [21,34]. Urea and SDS were thus able to destroy the respective hydrogen-bonds and hydrophobic interaction between the whey proteins and the two polyphenols. SDS addition in the assessed system thereby decreased the measured apparent binding constants. However, it was found unusually that urea addition led to enhanced apparent binding constants (Table 3). Urea thus showed an ability to enhance the WPI-polyphenol interactions, mainly due to the urea-induced protein unfolding. Whey proteins are characterized as water-soluble globulins, in which hydrogen-bonds play critical role to stabilize protein conformation. Urea destroyed these hydrogen-bonds efficiently, and thus unfolded these globulins in WPI. After then, WPI in the presence of urea showed higher affinity for galangin and genistein, because the resultant conformation change was beneficial to the polyphenol entrance and non-covalent protein-polyphenol binding. To the best of our knowledge, SDS and urea were not used in other studies to verify potential non-covalent interaction forces between the proteins and small molecules.

Protein affinity for phenol compounds are strongly affected by various factors such as the hydrophobicity and amino acid composition of proteins, as well as the molecular weights, chemical structures, and -OH numbers of phenol compounds [35,36]. Why β-lactoglobulin had higher affinity for galangin and genistein than α-lactalbumin can be explained by their different average hydrophobicity. It is addressed in a textbook that β-lactoglobulin has an average hydrophobicity value of 5.1 kJ/residue while α-lactalbumin shows an average hydrophobicity value of 4.7 kJ/residue [37]. Likely, the β-lactoglobulin-polyphenol complexes can yield stronger hydrophobic interaction than the α-lactalbumin-polyphenol complexes, based on essential knowledge of protein science. It was also reported that BSA had higher hydrophobicity than α-lactalbumin and lysozyme and thereby showed better affinity for chlorogenic acid [35], supporting the present result strongly. Moreover, proteins might provide similar or different binding sites for various polyphenols. When five polyphenols were bound to β-lactoglobulin, the revealed binding sites were different [15,38]. This protein provided a hydrophobic pocket consisting of 13 amino acid residues including Leu-39, Val-41, Leu-58, Lys-60, Glu-62, Lys-69, Ile-71, Ile-84, Asp-85, Ala-86, Asn-88, Asn-90, and Met-107 for catechin, or another 13 amino acid residues like Pro-38, Leu-39, Val-41, Ile-56, Leu-58, Lys-60, Lys-69, Ile-71, Ile-84, Asp-85, Asn-90, Phe-105, and Met-107 for epicatechin [15]. Clearly, only three amino acid residues were different, while other 10 residues were same. That is, β-lactoglobulin provided similar binding site to catechin and epicatechin. However, it was also detected that β-lactoglobulin provided a hydrophobic pocket with 22 residues (Ile-12, Val-15, Pro-38, Leu-39, Val-41, Val-43, Leu-46, Leu-54, Ile-56, Leu-58, Ile-71, Ala-73, Phe-82, Ile-84, Asn-90, Val-92, Val-94, Leu-103, Phe-105, Met-107, Gln-120, and Leu-122) for naringenin, 19 residues (Pro-38, Leu-39, Val-41, Tyr-42, Val-43, Leu-46, Leu-54, Ile-56, Leu-58, Lys-60, Lys-69, Ile-71, Ile-84, Asn-90, Val-92, Phe-105, Met-107, Gln-120, and Leu-122) for nobilettin, or 16 residues (Pro-38, Leu-39, Val-41, Val-43, Leu-46, Leu-54, Ile-56, Leu-58, Lys-69, Ile-71, Ile-84, Val-92, Leu-103, Phe-105, Met-107, and Leu-122) for tangeretin [38]. The three revealed pockets had different amino acid residues. These results thus supported that á-lactalbumin had different binding sites to galangin and genistein while the β-lactoglobulin could provide similar binding site to the two polyphenols. Overall, whey proteins might have non-specific binding sites to polyphenols.

Galangin always had higher binding ability to WPI than genistein although they are same in molecular weight and –OH group number, which might be arisen from different stereochemical features of the two polyphenols. Compared with galangin (the B-ring in C-2), genistein (the B-ring in C-3) shows a location isomerism of the B-ring, which conferred genistein with a weaker affinity for WPI. This conclusion was supported by a previous study [23], in which β-lactoglobulin showed higher affinity for apigenin (the B-ring in C-2) than genistein. Location isomerism of the B-ring was thus regarded to make contribution to the different affinities [23]. Moreover, flavonoid molecules with many –OH or other groups will rotate or twist the B-ring due to the steric hindrance, leading to destroyed planar stereochemical structure and weakened binding with DNA [39]. Two previous studies stressed that the molecules with near-planar structures were easier to enter the hydrophobic pockets of proteins [40,41]. Genistein has one –OH group in the B-ring, while galangin does not have any –OH group in the B-ring. Galangin thus possesses an ideal stereochemical structure. Due to its B-ring with less steric hindrance, galangin could rotate randomly to any ideal angle, and then interacted well with these amino acid residues in the hydrophobic pockets of proteins (Figure 4). However, genistein does not have these properties. The random rotation for the B-ring of galangin was suggested helping the formation of the WPI-galangin complex.

Changed secondary conformation of proteins could be induced by the interactions between proteins and small molecules. In the presence of β-carotene, caseins were found to have significant conformational change [29]. It was reported that the binding of a small molecule parecoxib with human serum albumin as well as the binding of tea polyphenols with milk proteins led to protein unfolding and conformational change [15,42]. When EGCG was bound into BSA, BSA’s secondary structure was also changed [28]. It was thus reasonable in the present study that the non-covalent interactions between WPI and galangin/genistein induced conformational changes for these proteins in WPI.

## 5. Conclusions

It is concluded that both galangin and genistein can non-covalently interact with WPI mainly through hydrophobic interaction, and WPI has higher affinity for galangin other than genistein. Both SDS and urea are able to decrease and increase the non-covalent interactions via destroying the hydrophobic interaction and breaking hydrogen-bonds to unfold proteins, and thus result in reduced and increased binding constants, respectively. The resultant non-covalent interactions lead to secondary conformational changes of the proteins in WPI, while the two polyphenols are likely to be bound at similar site in β-lactoglobulin but different sites in á-lactalbumin. The location isomerism and stereochemical structures of the two polyphenols as well as average hydrophobicity of α-lactalbumin and β-lactoglobulin have impact on the interactions, highlighting the critical roles of polyphenol structures and protein property in the non-covalent protein-polyphenol interactions, from a chemical point of view.

## Figures and Tables

**Table 1:** The linear equations, Stern–Volmer quenching constants (Ksv), and quenching rate constants (Kq) for the non-covalent WPI-galangin/genistein interactions at three temperatures.

Polyphenol	*T* (K)	Equation	Ksv (10^4^ L/mol)	Kq (10^12^ L/(mol·s))	*R* ^2^
Galangin	293	Y = 0.0838Q + 0.7249	8.38 ± 0.20^a^	8.38 ± 0.20^a^	0.959
	303	Y = 0.0891Q + 0.6836	8.91 ± 0.24^b^	8.91 ± 0.24^b^	0.953
	313	Y = 0.1046Q + 0.6227	10.46 ± 0.16^c^	10.46 ± 0.16^c^	0.955
Genistein	293	Y = 0.0417Q + 0.9484	4.17 ± 0.02^d^	4.17 ± 0.02^d^	0.993
	303	Y = 0.0496Q + 0.9059	4.96 ± 0.06^e^	4.96 ± 0.06^e^	0.974
	313	Y = 0.0581Q + 0.8899	5.81 ± 0.08^f^	5.81 ± 0.08^f^	0.989

Different lowercase letters after the data as superscripts in the same column indicate that the means of ANOVA using Duncan’s multiple comparison test differ significantly (*p* < 0.05). The critical R_0.01_ value for linear regression is 0.798 (*n* = 9).

**Table 2:** The apparent binding parameters (Ka) and three apparent thermodynamic parameters for the non-covalent WPI-galangin/genistein interactions at three temperatures.

Polyphenol	*T* (K)	*n*	Ka (10^5^ L/mol)	ΔH (kJ/mol)	ΔG (kJ/mol)	ΔS (J/(mol·K))
Galangin	293	1.22 ± 0.01	6.96 ± 0.65	12.5 ± 2.2	−(32.8 ± 2.5)	154.6 ± 13.8
	303	1.24 ± 0.04	9.03 ± 0.54		−(34.4 ± 2.6)	
	313	1.23 ± 0.01	9.64 ± 0.47		−(35.9 ± 2.4)	
Genistein	293	1.10 ± 0.02	1.08 ± 0.17	41.1 ± 1.8	−(28.1 ± 1.9)	236.4 ± 6.0
	303	1.12 ± 0.06	1.69 ± 0.95		−(30.5 ± 1.9)	
	313	1.15 ± 0.08	3.18 ± 0.76		−(32.9 ± 2.2)	

**Table 3:** Effects of sodium dodecyl sulfate (SDS) and urea on the apparent binding constants (Ka) for the non-covalent WPI-galangin/genistein interactions at 293 K.

Protein-Polyphenol Complex	SDS/Urea Addition	Ka (L/mol)
WPI-galangin	SDS	(2.24 ± 0.52) × 10^5^
	Urea	(8.95 ± 0.42) × 10^5^
WPI-genistein	SDS	(3.93 ± 0.23) × 10^4^
	Urea	(1.59 ± 0.22) × 10^5^

**Table 4:** The energy transfer parameters for the non-covalent WPI-galangin/genistein interactions.

Protein-Polyphenol Complex	J (cm3·L/mol)	R0 (nm)	E	r (nm)
WPI-galangin	5.30×10^-15^	2.41	0.542	2.34
WPI-genistein	1.38×10^-16^	1.31	0.417	1.39

**Table 5:** 3D fluorescence spectral characteristics of WPI or the WPI-galangin and WPI-genistein complexes.

Peak Parameter		WPI	WPI-Galangin	WPI-Genistein
Peak I	Peak position λ_ex_/λ_em_ (nm/nm)	240/340	240/345	240/345
	Fluorescence intensity	311.4	130.5	115.7
Peak II	Peak position λ_ex_/λ_em_ (nm/nm)	280/340	280/345	280/345
	Fluorescence intensity	2327	639.6	1028

**Table 6:** The amino acid residues, H-bond number, and interaction energy ΔG values involved in the non-covalent interaction of galangin/genistein with β-lactoglobulin/α-lactalbumin.

Protein and Polyphenol	Involved Residue	H-Bond Number	ΔG (kJ/mol)
β-Lactoglobulin and galangin	Leu-10, Asp-11, Ile-12, Lys-75, Thr-76, Lys-77, Ile-78, Pro-79, Ala-80, Val-81, Phe-82	0	−30.06
β-Lactoglobulin and genistein	Leu-10, Asp-11, Ile-12, Gln-13, Lys-75, Thr-76, Ile-78, Pro-79, Ala-80, Val-81, Phe-82	0	−29.39
α-Lactalbumin and galangin	Pro-109, Leu-110, Cys-111, Ser-112 *, Asp-113, Lys-114, Leu-115, Gln-117, Trp-118	1	−25.62
α-Lactalbumin and genistein	Gln-2, Leu-3, Thr-4, Lys-5, Phe-31, Tyr-36, Gln-117, Trp-118	0	−27.42

* Hydrogen bonding with this residue.
